# *In Vitro* Reconstitution of Yeast tUTP/UTP A and UTP B Subcomplexes Provides New Insights into Their Modular Architecture

**DOI:** 10.1371/journal.pone.0114898

**Published:** 2014-12-12

**Authors:** Gisela Pöll, Shuang Li, Uli Ohmayer, Thomas Hierlmeier, Philipp Milkereit, Jorge Perez-Fernandez

**Affiliations:** Lehrstuhl für Biochemie III, Universität Regensburg, Regensburg, Germany; Université Libre de Bruxelles. BELGIQUE, Belgium

## Abstract

Eukaryotic ribosome biogenesis is a multistep process involving more than 150 biogenesis factors, which interact transiently with pre-ribosomal particles to promote their maturation. Some of these auxiliary proteins have been isolated in complexes found separate from the ribosomal environment. Among them, are 3 large UTP subcomplexes containing 6 or 7 protein subunits which are involved in the early steps of ribosome biogenesis. The composition of the UTP subcomplexes and the network of binary interactions between protein subunits have been analyzed previously. To obtain further insights into the structural and biochemical properties of UTP subcomplexes, we established a heterologous expression system to allow reconstitution of the yeast tUTP/UTP A and UTP B subcomplexes from their candidate subunits. The results of a series of reconstitution experiments involving different combinations of protein subunits are in good agreement with most of the previously observed binary interactions. Moreover, in combination with additional biochemical analyses, several stable building blocks of the UTP subcomplexes were identified. Based on these findings, we present a refined model of the tUTP/UTP A and UTP B architecture.

## Introduction

Eukaryotic ribosome biogenesis is a complex process [1] which involves synthesis, processing and folding of the four ribosomal RNAs (rRNAs), and the stable assembly of ∼80 ribosomal proteins. Furthermore, in *S. cerevisiae* (hereafter referred to as yeast), more than 150 non-ribosomal proteins, termed biogenesis factors, and 70 small nucleolar RNAs interact transiently with pre-ribosomal particles in the course of ribosome maturation [2]–[4]. Although recent studies have made progress to elucidate the different stages of ribosomal assembly, the detailed molecular function of most of the ribosome biogenesis factors has yet to be determined.

More than 10 years ago, analysis with mass spectrometry (MS) of the small subunit (SSU) processome or 90S pre-ribosome, the earliest pre-ribosomal particle that can be isolated, identified approximately 40 ribosome biogenesis factors [5], [6]. Due to the association of those proteins with the U3 snoRNA, 20 of these factors were named U three proteins (Utps). Further analysis in yeast revealed that several Utps could be grouped into the protein subcomplexes termed UTP A and UTP B [7].

UTP A was isolated as a protein complex composed of Utp4, Utp8, Utp9, Utp10, Utp15, Pol5 and Nan1 [7]. With the exception of Pol5, yeast UTP A components, together with Utp5, have been suggested to be required for optimal rDNA transcription and, therefore, have been designated as tUtps (transcription Utps) [8], [9]. The observed interactions between some of these tUtps, as well as their common function in promoting transcription, suggested the existence of a functional protein subcomplex, which is now known as the tUTP subcomplex [8]. Nevertheless, tUTP and UTP A have been proposed to be the same protein complex despite the difference in molecular composition in regards to the presence of Utp5 or Pol5, respectively [8], [10]. In this work, we have chosen to refer to this protein complex as tUTP.

UTP B was characterized as a stoichiometric, six-protein complex consisting of Utp6, Utp13, Utp18, Utp21, Dip2 and Pwp2 [7]. In good agreement, UTP B has been purified from yeast extracts as an isolated particle under conditions which impaired its binding to pre-rRNA [11].

Several approaches have been used in order to ascertain the architecture and organization of the UTP subcomplexes. For example, yeast two-hybrid based approaches were able to pinpoint the physical interactions between different subcomplex components [12]–[14]. Additionally, identification of the cross-linked peptides via MS analysis from chemical crosslinking of reconstituted yeast UTP B, provided valuable initial structural information [15].

In this work we analyzed the architectural and biochemical properties of the yeast subcomplexes tUTP/UTP A and UTP B with the use of a flexible heterologous expression system to reconstitute the complexes from the defined candidate protein subunits. By combining this approach with further biochemical analyses, we identified several architectural building blocks of tUTP and UTP B, which might represent intermediate states during the assembly or disassembly of UTP subcomplexes.

## Materials and Methods

### Generation of recombinant baculoviruses, SF21 insect cell culture, and heterologous protein expression

Recombinant baculoviruses encoding combinations of the proteins of the tUTP or UTP B subcomplexes were constructed using the MultiBac System as previously described [16], [17]. Coding regions of the respective yeast genes were amplified by PCR and inserted into the plasmids pUCDM, pFL, pSPL or derivatives thereof by standard cloning procedures [18]. The oligonucleotides and plasmids used are listed in Tables 1 and 2, respectively. Fusion plasmids containing different combinations of genes were obtained by *in vitro* Cre-Lox recombination of the respective plasmids using Cre Recombinase (New England Biolabs, Inc., Ipswich, MA, USA). The fusion plasmids were integrated into the viral genome by transformation into DH10MultiBac-EYFP *E. coli* cells. Recombinant bacmid DNA was isolated and transfected into adherently growing SF21 insect cells using FuGeneHD transfection reagent (Promega Corp., Madison, WI, USA) to generate recombinant baculoviruses (V0 stock). V0 viruses were amplified in 50 mL SF21 cultures over a period of 3–5 days (V1 stock), which was subsequently used for expression of the recombinant proteins. Aliquots of 5 mL of V1 virus stock were used to infect 200 mL SF21 cell culture (1×10^6^ cells mL^−1^) in 1 L Erlenmeyer flasks and incubated for 48 h at 27°C. Cells were harvested in aliquots of 50×10^6^ cells by centrifugation (130×*g*, 10 min, room temperature). Finally, cell pellets were frozen in liquid nitrogen and stored at −20°C.

**Table 1:** Oligonucleotides: Oligonucleotides used for cloning of Utp genes are described. Database numbers, gene amplified and sequence are indicated.

Database Nr.	Gene amplified	Sequence 5′ to 3′
2517	Primer for cloning of TAP phused genes with Nsi1 sequence.	TTTTTTATGCATTCAGGTTGACTTCCCCGCGG
2718	primer for *UTP15* cloning in Multibac Vectors.	TTGTTGGAATTCATGGATTACAAGGATGACGACGATAAGGCTGCAATGTCGACTGCTAGGCCTAGA
2719	primer for *UTP15* cloning in Multibac Vectors.	TTGTTGCTGCAGTTAACTCGTTAAAAGTTGAAGCATA
2720	primer for *UTP9* cloning in Multibac Vectors.	TTGTTGCTCGAGATGGGCTCCTCTTTGGATTT
2721	primer for *UTP9* cloning in Multibac Vectors.	TTGTTGGCTAGCTCAATCTTTGTATTCCGATGC
2722	primer for *UTP4* cloning in Multibac Vectors.	TTGTTGCTCGAGATGAGCTCATCGCTACTTTCAG
2723	primer for *UTP5* cloning in Multibac Vectors.	TTGTTGGTCGACATGGATTCTCCTGTTCTACAGTC
2724	primer for *UTP5* cloning in Multibac Vectors.	TTGTTGCTGCAGCTATTCCATCTCAACGTCACTATATC
2725	primer for *UTP10* cloning in Multibac Vectors.	TTGTTGCTCGAGATGTCTTCGTTGAGTGACCAAT
2726	primer for *UTP10* cloning in Multibac Vectors.	TTGTTGGCTAGCCTAATCTAAATACCTATCAAAAGGTTC
2727	primer for *NAN1* cloning in Multibac Vectors.	TTGTTGGTCGACATGACGCAATCCCTAGGTATC
2728	primer for *NAN1* cloning in Multibac Vectors.	TTGTTGCTGCAGCTATGTTAATACTTTCATCACACGATC
2729	primer for *UTP8* cloning in Multibac Vectors.	TTGTTGGGATCCATGCCATCCCTGTCTCAACC
2730	primer for *UTP8* cloning in Multibac Vectors.	TTGTTGGTCGACTCAAATGTCCAAGTATTCCATGG
3156	primer for *UTP13* cloning in Multibac Vectors.	TTGTTGCCCGGGATGGATCTGAAAACCTCATATAAAG
3157	primer for *UTP13* cloning in Multibac Vectors.	TTGTTGCTCGAGCTAGAATAGCTTATCCATTTCCACT
3158	primer for *UTP12* cloning in Multibac Vectors.	TTGTTGGTCGACATGGTCAAATCATACCAACGTT
3159	primer for *UTP12* cloning in Multibac Vectors.	TTGTTGCTGCAGTTATATAACGGTCCCGAAAACT
3160	primer for *UTP18* cloning in Multibac Vectors.	TTGTTGGGATCCATGACAATGGCAACAACCG
3161	primer for *UTP18* cloning in Multibac Vectors.	TTGTTGCTGCAGTTAGTAGTGGTTTAATTTCCAGAGC
3162	primer for *PWP2* cloning in Multibac Vectors.	TTGTTGCCCGGGATGAAATCCGATTTCAAGTTCTCT
3163	Primer for cloning of TAP fused genes with XhoI sequence.	TTGTTGCTCGAGTCAGGTTGACTTCCCCGC
3164	primer for *PWP2* cloning in Multibac Vectors.	TTGTTGCTCGAGTCAAGGAAGCTCTTTCTCATTTT
3165	primer for *UTP6* cloning in Multibac Vectors.	TTGTTGGTCGACATGTCGAAGACAAGATACTATTTGG
3166	primer for *UTP6* cloning in Multibac Vectors.	TTGTTGCTGCAGTTAAAGTTTGCTGATAATTAAATCTAGAA
3167	primer for *UTP21* cloning in Multibac Vectors.	TTGTTGCCCGGGATGTCTATCGACTTGAAAAAAAGAAA
3168	primer for *UTP21* cloning in Multibac Vectors.	TTGTTGCTCGAGTCACGCGGTGGTCACAAA
3236	Primer for cloning of TAP fused genes with SphI sequence.	TTGTTGGCATGCTCACTGATGATTCGCGTCTACTT
3239	primer for *UTP4* cloning in Multibac Vectors.	TTGTTGATGCATTCAAAACACTAACTTTGGTTGAATAA

**Table 2:** Plasmids: Description of plasmids used in this work. Database Number, plasmid backbone used to clone the indicated genes is specified. Original References for previously used plasmids are indicated. When required, plasmids used during the recombination reaction are also indicated.

Database Nr.	Plasmid Backbone	Genes cloned	Refs. Plasmid used in the recombination reaction
K1127	pUCDM	-	[17]
K1130	pFL	-	[17]
K1212	pFL-FLAG	-	[19]
K1502	pSPL-3xHA	-	[19]
K1670	pFL-FLAG	*UTP15*	This work.
K1671	pFL-FLAG	*UTP15, UTP9*	This work.
K1672	pUCDM	*UTP4-*TAP	This work.
K1673	pUCDM	*UTP5*	This work.
K1682	pUCDM	*UTP5, UTP4-*TAP	This work.
K1684	pSPL-3xHA	*NAN1, UTP10*	This work.
K1685	pFL-	*UTP5, UTP9, UTP15-*FLAG	This work. Amplification module.
K1721	pFL-	*UTP4-*TAP, *UTP5*	This work
K1978	pFL-FLAG	*UTP12, UTP13*	This work.
K1979	pUCDM	*UTP18*	This work.
K1980	pUCDM	*UTP18, PWP2-*TAP	This work.
K1981	pUCDM	*UTP18, PWP2*	This work.
K1982	pFL-	*UTP6-*HA	This work
K1983	pSPL-3xHA	*UTP6-*HA, *UTP21*	This work.
K1986	pFL-	*UTP6-*HA, *UTP21-*TAP	This work. K1130+K2122
K1987	pFL-	*UTP6-*HA, *UTP18, UTP21, PWP2-*TAP	This work. K1980+K1983
K1991	pFL-	*UTP6-*HA, *UTP12-*FLAG, *UTP13, UTP18, UTP21, PWP2-*TAP	This work. K1978+K1980+K1983
K1992	pFL-	*UTP6-*HA, *UTP12-*FLAG, *UTP13, UTP18, UTP21, PWP2*	This work. K1978+K1981+K1983
K1997	pUCDM	*UTP5, UTP4*	This work.
K1999	pFL-FLAG	*NAN1, UTP10*	This work.
K2000	pFL-	*UTP4, UTP5, UTP8, UTP9, UTP10, UTP15-*FLAG, *NAN1-*HA	This work. K1684+K1685+K1997
K2122	pSPL-3xHA	*UTP6, UTP21-*TAP	This work.
K2123	pFL-	*UTP4-*TAP, *UTP5, UTP8, UTP9, UTP15-*FLAG	This work. K1682+K1685
K2124	pFL-	*UTP5, UTP15-*FLAG	This work. K1670+K1673
K2126	pFL-	*UTP5, UTP8, UTP9, UTP15-*FLAG	This work. K1673+K1685
K2134	pFL-	*UTP6-*HA, *UTP18, PWP2-*TAP	K1130+K1980+K1982
K2135	pFL-	*UTP6-*HA, *UTP21-*TAP	This work. K1130+K2122
K2136	pFL-	*UTP6-*HA, *UTP18, UTP21-*TAP	This work. K1986+K1979
K2137	pFL-	*UTP6-*HA, *UTP18, UTP21-*TAP, *PWP2*	This work. K1130+K1980+K1983
K2138	pFL-	*UTP18*	This work. K1130+K1979
K2139	pFL-	*UTP6-*HA, *UTP18*	This work. K2138+K1982
K2204	pFL-FLAG	*UTP8, UTP9,*	This work.
K2209	pFL-	*UTP4-*TAP, *UTP5, UTP15-*FLAG	This work. K1670+K1682
K2212	pFL-	*UTP4-*TAP, *UTP8-*FLAG, *UTP9*	This work. K1672+K2204

### Affinity purification of recombinantly expressed fusion proteins

Cell pellets derived from 50×10^6^ infected cells were processed as previously described to generate cellular lysates [19]. When specified, an ultracentrifugation step before the affinity purification was done using rotor TFT55.38 (Kontron Instruments, Germany) at 200000×*g* for 1 h at 4°C in a Optima L-80 XP ultracentrifuge (Beckmann Coulter, Krefeld, Germany). For the one–step purifications, 150 µL of slurry of the corresponding resin for the bait protein was pre-equilibrated in buffer A100+ (20 mM Tris–HCl pH 8, 100 mM KCl, 5 mM Mg(OAc)_2_, 2 mM Benzamidine, 1 mM PMSF, 0.5% Triton X-100, 0.1% Tween-20), and subsequently incubated with the clarified cell extracts for 2 h at 4°C on a turning wheel. The supernatant was removed after centrifugation (4°C, 1 min, 130×*g*), and the resin was washed with buffer A100+ in batch mode (3×10 mL, 3×1 mL). To elute the different fusion proteins, the resin was incubated with 100 µL buffer A100+, and relevant elution protocols were applied. In the case of FLAG-tagged proteins, Anti-FLAG M2 Affinity Gel (Sigma-Aldrich, St. Louis, MO, USA) resin was used, and the elution was performed in the presence of 300 µg mL^−1^ FLAG peptide (Sigma-Aldrich) with a 2 h at 4°C-incubation on a turning wheel. In the case of TAP-tagged proteins, IgG Sepharose 6 Fast Flow (GE Healthcare) resin was used, and the proteins were eluted in the presence of 1 µg mL^−1^ of 6xHis-tagged recombinant TEV protease. For HA-tagged proteins, anti-HA Affinity Matrix (Roche, Basel, Switzerland) resin was used, and 500 µg mL^−1^ HA-peptide was applied during elution. Finally, the resin beads were removed from the eluate by centrifugation (4°C, 1 min, 16000×*g*) through a MobiCol microspin column (MoBiTec, Goettingen, Germany).

Affinity-purified protein complexes were analyzed using the Smart System (Pharmacia Biotech) and a Superose6 PC 3.2/30 gel filtration column (GE Healthcare) equilibrated with buffer A100 (A100+ lacking protease inhibitors and Triton X-100) at a flow rate of 20 µL min^−1^ at 4°C. Fractionation (20×100 µL fractions) was started 35 min after sample injection.

For the two–step purifications, the procedure was similar to the one described for the one-step purification except that 2×10^8^ infected cells were used. The eluate from the first purification step was used in a second affinity purification performed with the corresponding resin (as described for the one-step purification).

### Western blotting (WB) analysis

Expression and purification of proteins from SF21 insect cells were monitored by WB. FLAG-tag and HA-tag fusion proteins were detected with anti-FLAG (L5, Agilent, Santa Clara, CA, USA) and anti-HA antibodies (3F10, Roche), in combination with an anti-rat HRP-coupled secondary antibody (112-035-068, Jackson Immuno Research, West Grove, PA, USA). TAP-tag fusion proteins were detected with PAP detection reagent (P1291, Sigma-Aldrich) or with anti-CBP (Calmodulin Binding Protein) antibody (sc-32998, Santa Cruz Biotechnology, Inc, Dallas, TX, USA) combined with an anti-goat HRP-coupled secondary antibody (sc-2020, Santa Cruz Biotechnology, Inc.). Protein signals were visualized using BM Chemiluminescence Western-blotting reagent (Roche) and an LAS-3000 Image Reader (Fujifilm).

### Gel-based mass spectrometric analysis of the proteins

Mass spectrometric analysis of Coomassie Blue-stained protein bands was done as previously described [20]. Peptide mass fingerprinting and tandem MS (MS/MS) analyses were performed in a 4800 Proteomics Analyzer MALDI-TOF/TOF instrument (ABI, Grand Island, NY USA), operated in positive-ion reflector mode. The data were evaluated by searching the NCBI non-redundant (nr) protein sequence database using the Mascot module implemented in the GPS Explorer, version 3.5, software (ABI).

## Results

### Yeast tUTP and UTP B subcomplexes can be assembled in SF21 cells

Protein-protein binary interactions between individual components of the yeast subcomplexes tUTP or UTP B were previously characterized via yeast two-hybrid analyses [12], [14]. In the case of UTP B, further insights on the potential architectural features were obtained by *in vitro* cross-linking of the purified recombinant subcomplex and subsequent MS analysis [15]. To complement the published data, this study aimed to reconstitute yeast subcomplexes tUTP and UTP B using insect cells as a heterologous expression system. Candidate components for either tUTP or UTP B were co-expressed in SF21 insect cells infected with respective recombinant baculoviruses [16], [17]. For each UTP subcomplex, several subunits were expressed in fusion with different epitope tags to allow the use of two-step affinity purification strategies. The protein composition of these purified complexes was analyzed by SDS-PAGE followed by MS analyses of single protein bands. Finally, the integrity of the purified protein complexes was verified by gel filtration.

**Figure 1:**
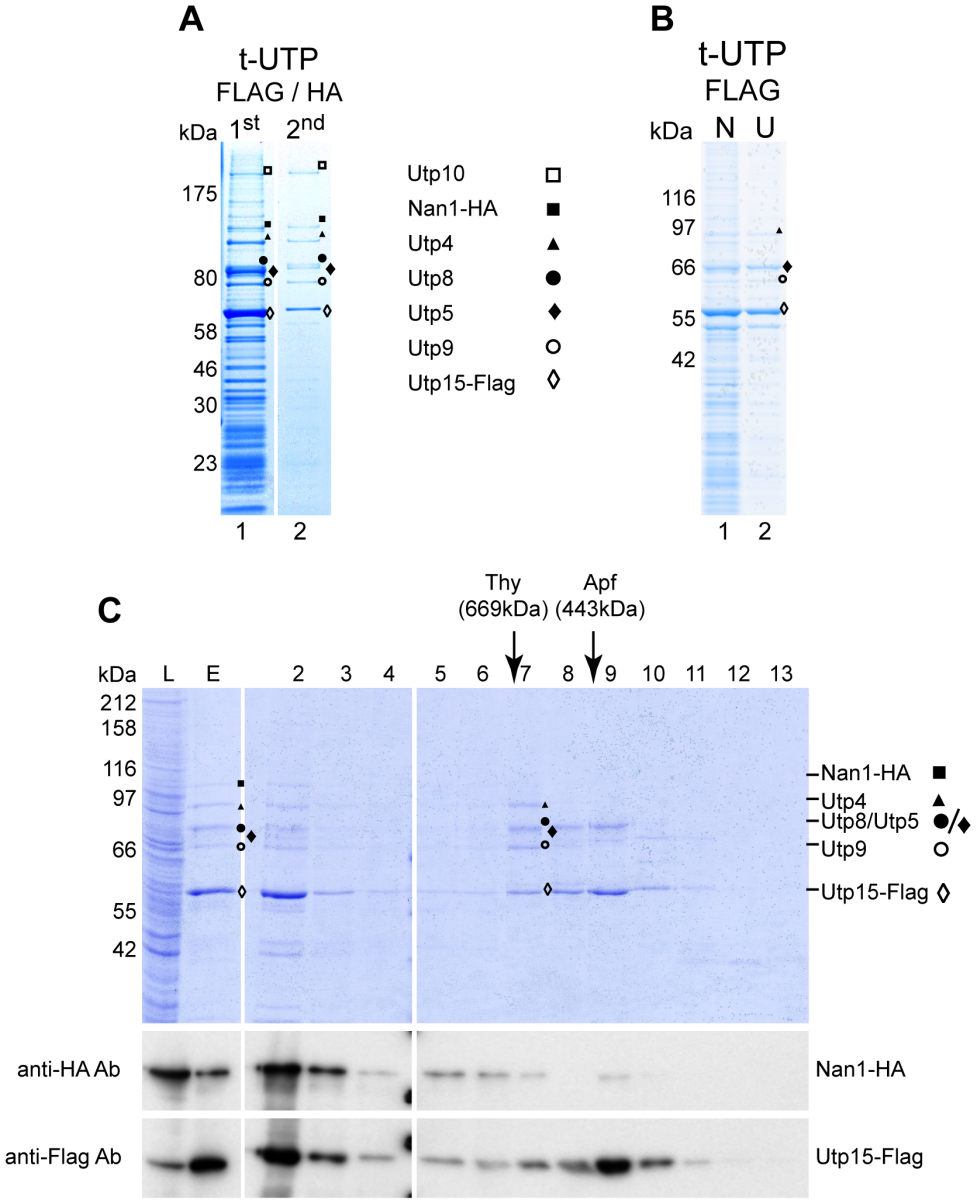
Yeast tUTP subcomplex reconstitution in insect cells. All candidate tUTP components were co-expressed in SF21 insect cells infected with baculoviruses containing bacmid K2000. Proteins identified by MS analysis are indicated as Nan1, ▪; Utp10, □; Utp4, ▴; Utp5, ♦; Utp8, •; Utp9, ○ and Utp15, ◊. (**A**) Two-step affinity purification using two different bait proteins. Lysates of 2×10^8^ infected cells were used in the first affinity purification step to purify Utp15-FLAG-containing component with anti-FLAG affinity matrix which were eluted with the FLAG peptide (Lane 1). 90% of the eluted material was used for the second affinity purification step with anti-HA affinity matrix to purify Nan1-HA containing components, which were eluted with the HA peptide (Lane 2). The composition of both eluates was analyzed on a 4–12% gradient SDS-PAGE, stained with Coomassie Blue, and the protein content of the indicated bands was identified by MS analysis. (**B**) Lysates of 8×10^7^ SF21 cells infected with baculovirus K2000 were cleared by low-speed centrifugation as described (N samples) and half of the sample was further cleared by ultracentrifugation (200000×*g*, 1 h, 4°C, U samples). Utp15-FLAG-containing components were purified from both lysates using anti-FLAG affinity matrix and eluted with the FLAG peptide. The eluted material (10%) was analyzed on a 4–12% gradient SDS-PAGE, stained with Coomassie Blue, and the protein content of the indicated bands was identified by MS analysis. (**C**) Utp15-FLAG-containing components were purified from lysates of 4×10^7^ infected cells using anti-FLAG affinity matrix and eluted with the FLAG peptide. Half of the eluate was fractionated on a Superose 6 gel filtration column. Aliquots of the lysate (L, 0,03%), the eluate (E, 10%) and the fractions (2–13; 15%) were analyzed by SDS-PAGE (upper panel) and by WB using antibodies against HA (middle panel) or FLAG (lower panel) epitopes. Elution of marker proteins in independent gel filtration runs are indicated at the top. Correct identification of the corresponding protein by MS analysis is indicated.

Candidate yeast tUTP components (Utp4, Utp5, Utp8, Utp9, Utp10, Utp15 and Nan1) were co-expressed in SF21 insect cells with Utp15 and Nan1, tagged with FLAG and HA epitopes, respectively. One-step affinity purification from cellular extracts of Utp15-FLAG significantly enriched all selected tUTP components (Fig. 1A, Lane 1). However, several additional, unidentified bands were observed mainly in the lower molecular weight range. The FLAG-purified complex was subjected to a second affinity purification using Nan1-HA as the bait protein. This second affinity purification step led to a strong reduction in the low molecular weight contaminants, whereas all known tUTP components were still retained (Fig. 1A, Lane 2). Most of these contaminants were removed when cellular extracts were clarified by ultracentrifugation prior to the one-step affinity purification (Fig. 1B, compare lanes 1 and 2).

Analysis of the gel filtration elution profiles for the Utp15-FLAG-purified complexes revealed that Utp15-FLAG co-eluted with Utp4, Utp5, Utp8, and Utp9 in fraction 7 (Fig. 1C, Fraction 7). These results strongly indicate that these five proteins form a defined multi-protein complex, which was designated as the tUTP pentamer. Western blot analysis of the gel filtration fractionation confirmed the co-elution of Nan1-HA with Utp15-FLAG in fractions corresponding, by molecular weight, to a fully reconstituted tUTP (Fig. 1C, middle and lower panels, Fractions 5 and 6). Furthermore, the WB analysis also showed the presence of smaller complexes containing Nan1-HA (Fig. 1C, middle panel, Fraction 9), which could indicate a loose association of Nan1 to the tUTP pentamer. Utp10, however, could not be observed in this analysis. In agreement with a weak association, neither Nan1 nor Utp10 could be detected in Utp15-FLAG-associated complexes when the cellular extracts were cleared by ultracentrifugaton (Fig. 1B). Altogether, these results showed that a pentameric core complex of recombinant yeast tUTP components (Utp4, Utp5, Utp8, Utp9 and Utp15) can be reconstituted in a heterologous expression system. In the experimental conditions used, the proteins Nan1 and Utp10 appeared to be only loosely associated with the tUTP pentamer.

**Figure 2:**
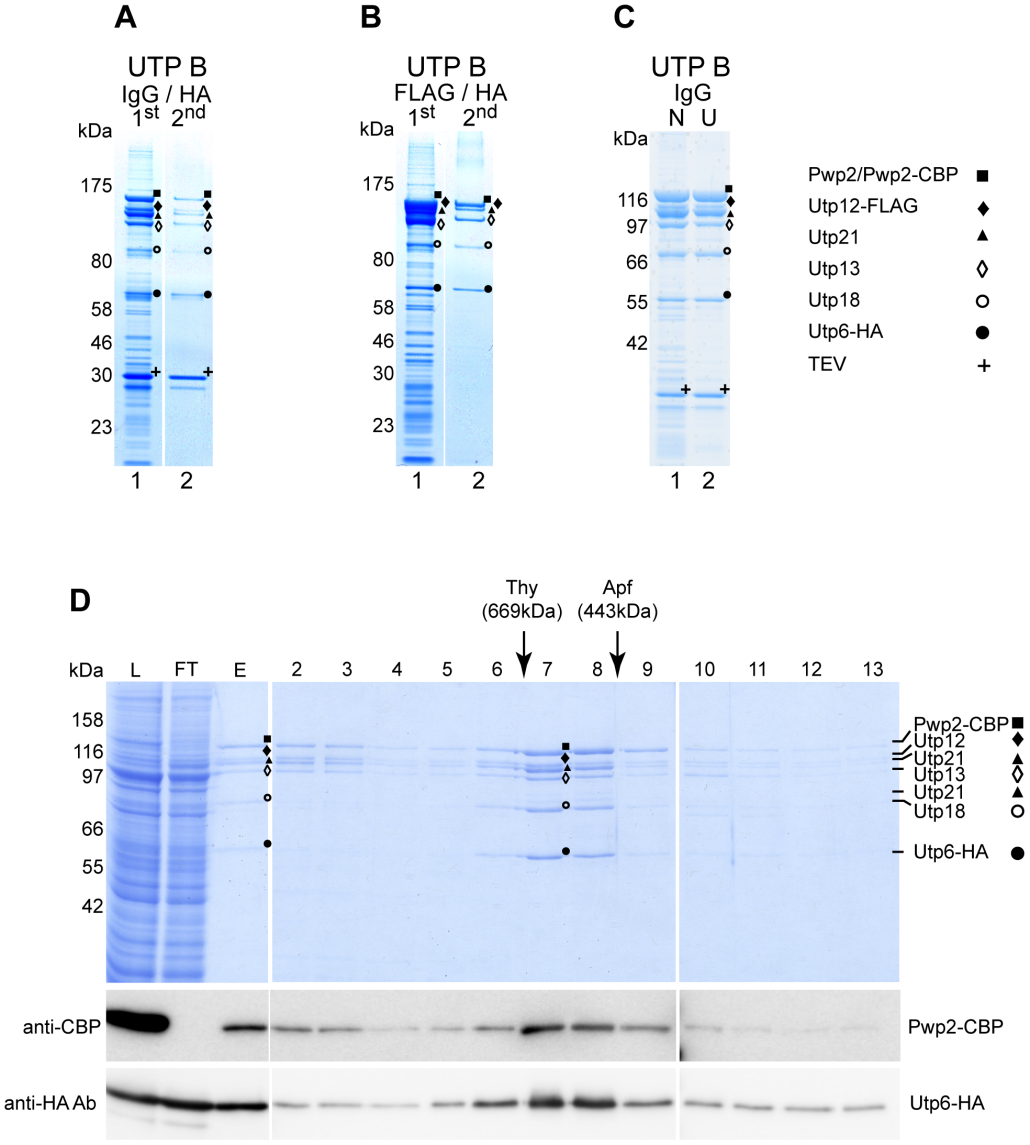
Yeast UTP B subcomplex reconstitution in insect cells. All selected UTP B components were co-expressed in SF21 insect cells infected with baculoviruses containing the bacmids K1991 or K1992. The protein content of the indicated bands was identified by MS and are indicated as Pwp2, ▪; Utp6, •; Utp12, ♦; Utp13, ◊; Utp18, ○ and Utp21, ▴. (**A**) Lysates of 2×10^8^ cells infected with K1991were used for two-step affinity purification. Pwp2-TAP was used as the bait protein in the first affinity purification step with IgG-coupled Sepharose resin, and Pwp2-containing components were eluted with TEV protease (Lane 1). Utp6-HA-containing components were purified from 90% of the first elution sample using anti-HA affinity matrix, followed by elution with the HA peptide (Lane 2). The composition of the eluate (5% each) was analyzed on a 4–12% gradient SDS-PAGE, stained with Coomassie Blue, and analyzed by MS. (**B**) Lysates of 2×10^8^ cells infected with K1992 were used for two-step affinity purification. Utp12-FLAG was purified with anti-FLAG affinity matrix and eluted with the FLAG peptide during the first affinity purification step (Lane 1). A 90% aliquot of the eluted material was used to purify Utp6-HA-containing components with anti-HA affinity matrix, followed by elution with the HA peptide (Lane 2). The composition of both eluates (5%) was analyzed on a 4–12% gradient SDS-PAGE, stained with Coomassie Blue, and analyzed by MS. (**C**) Lysates of 8×10^7^ SF21 cells infected with bacmid K1991 were cleared by the low-speed centrifugation described in the normal protocol (N samples), and half was further cleared by ultracentrifugation (200000×*g*, 1 h, 4°C, U samples). Pwp2-TAP-containing components were purified from both lysates using IgG-coupled Sepharose resin and eluted with TEV protease. A 10% aliquot of the eluted material was analyzed on a 4-12% gradient SDS-PAGE, stained with Coomassie Blue, and analyzed with MS. (**D**) Pwp2-TAP-containing components were purified from lysates of 4×10^7^ infected cells (K1991) using IgG-coupled Sepharose resin and TEV elution. Half of the eluate was fractionated on a Superose 6 gel filtration column. Aliquots of the lysate (L, 0,03%), flow through from the first purification (FT, 0,03%), the eluate from the affinity column (E, 10%), and the fractions from the gel filtration column (2–13; 15%) were analyzed by SDS-PAGE (upper panel) and WB with antibodies against CBP (middle panel) or HA (lower panel) epitopes. Elution of marker proteins in independent gel filtration runs are indicated at the top. Correct identification by MS analysis of the corresponding protein is indicated.

In order to characterize the components of the yeast subcomplex UTP B, cell lysates of SF21 insect cells co-expressing Pwp2-TAP, Utp6-HA, Utp12-FLAG, Utp13, Utp18, and Utp21 were subjected to Pwp2-TAP affinity purification via IgG Sepharose. MS analysis identified all six recombinant proteins in the eluate, as well as, the TEV protease used for elution (Fig. 2A, Lane 1). Similarly, after co-expression of Pwp2, Utp6-HA, Utp12-FLAG, Utp13, Utp18, and Utp21 and FLAG affinity purification from the cell lysates, all selected proteins were detected through MS analysis of the eluate (Fig. 2B, Lane 1). In both cases, several unidentified SDS-PAGE bands were observed migrating mainly in the lower molecular weight range. Pwp2-TAP and Utp12-FLAG affinity-purified complexes were subjected to a second affinity purification step using Utp6-HA as the bait protein. All co-expressed components were identified by MS analysis in the respective eluates, and all were confirmed to be present in stoichiometric amounts by SDS-PAGE analysis (Figs. 2A and B, Lane 2). Despite the residual amounts of TEV protease detected in the final eluate, the second affinity purification step led to a significant reduction of low molecular weight contaminants. As was observed for tUTP, the contaminants were also diminished if the cellular extracts were cleared by ultracentrifugation before the one-step affinity purification procedure (Fig. 2C, compare lanes 1 and 2).

Consistent with the reconstitution of a defined yeast multi-protein complex in insect cells, all UTP B components (Pwp2-Utp21-Utp12-Utp13-Utp6-Utp18) purified via Pwp2-TAP, co-migrated in the gel filtration elution profile with an apparent molecular weight of around 670 kDa (Fig. 2D, Fractions 7 and 8). This estimated molecular weight closely matches the theoretical mass of 550 kDa, expected for a fully reconstituted, hexameric UTP B subcomplex. Interestingly, Utp12-FLAG and Utp13 seemed to be partially underrepresented in fraction 8, when compared to fractions 6 and 7. This finding suggests the formation of a partially assembled UTP B subcomplex lacking these two proteins (Fig. 2D, upper panel, compare intensity of Coomassie staining of proteins in all fractions). In summary, these experiments show that the yeast UTP B complex can be reconstituted from recombinant proteins expressed in insect cells. Furthermore, the results suggest the formation of a stable UTP B core-complex, composed of Pwp2, Utp6, Utp18 and Utp21, to which Utp12 and Utp13 can associate.

Altogether, we conclude that the recombinant production of either tUTP or UTP B in insect cells allowed the recovery of highly purified protein complexes. These complexes seem to contain stoichiometric amounts of their known protein components. Thus, tUTP and UTP B can be formed in the absence of any other yeast factors (see discussion).

### Identification of the building blocks of the yeast tUTP subcomplex

**Figure 3:**
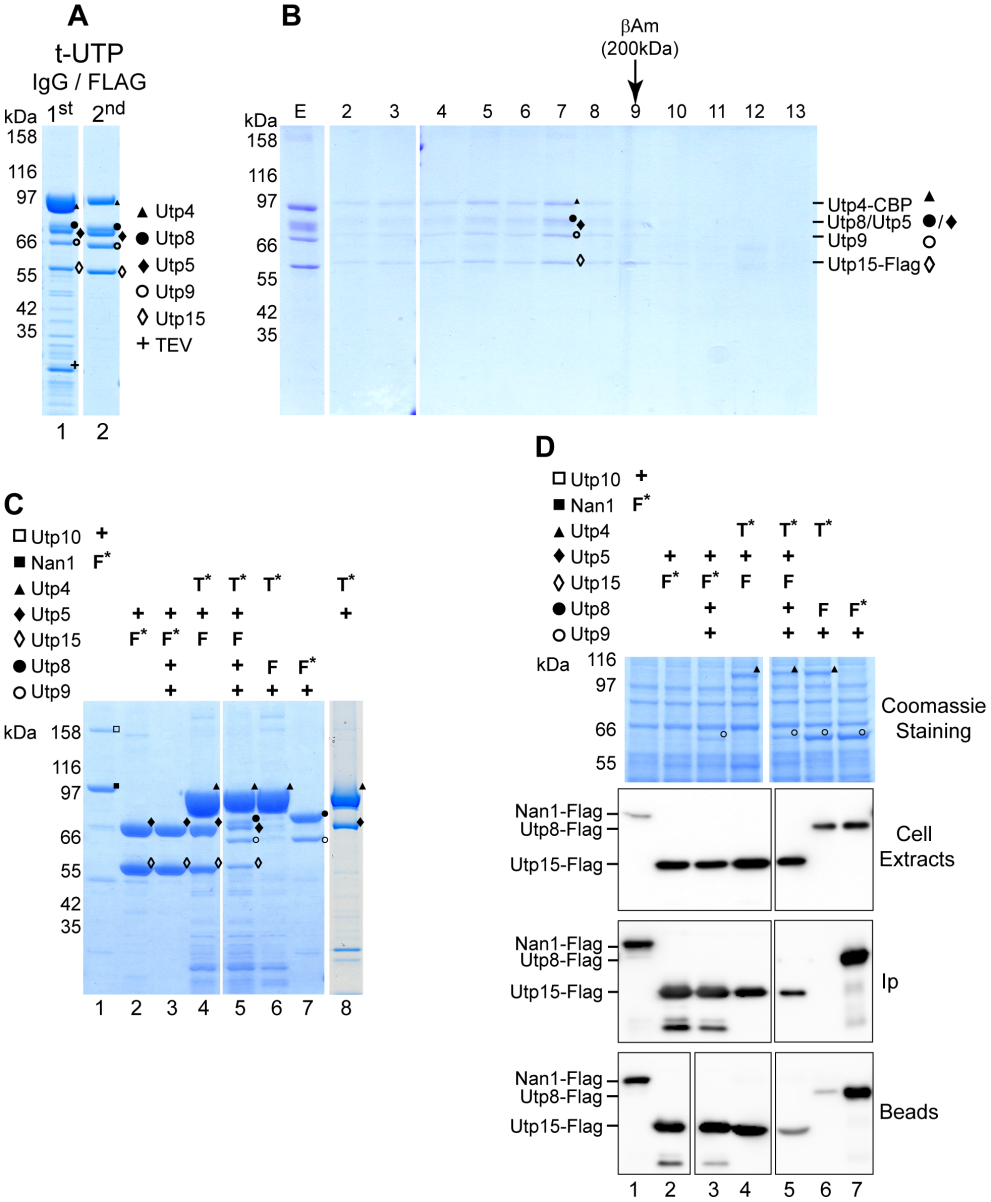
Indentification of different tUTP building blocks. Tagged proteins were purified from cell extracts containing different tUTP components in one or two step affinity purifications. Correct identification by MS analysis of the corresponding protein is indicated as Nan1, ▪; Utp10, □; Utp4, ▴; Utp5, ♦; Utp8, •; Utp9, ○ and Utp15, ◊. (**A**) Utp4-TAP, Utp5, Utp8, Utp9 and Utp15-FLAG were co-expressed in SF21 insect cells infected with a baculovirus containing the bacmid K2123. Utp4-TAP protein was purified from lysates of 2×10^8^ infected cells with IgG-coupled Sepharose resin and eluted with TEV protease (Lane 1). The eluted material (80%) was used for the second affinity purification step with anti-FLAG affinity matrix to purify Utp15-FLAG-containing components, which were then eluted with the FLAG peptide (Lane 2). In both cases, 10% of the eluted fraction was analyzed on a 4–12% gradient SDS-PAGE, stained with Coomassie Blue, and analyzed by MS. (**B**) The eluted material (30%) from the second affinity purification (see part A) was fractionated on a Superose 6 gel filtration column. Samples of the affinity elution (E, 10%) and fractions from the gel filtration column (2–13; 15%) were analyzed by SDS-PAGE (upper panel). The elution of protein standards from independent gel filtration runs are indicated at the top. (**C**) The indicated combinations of proteins were co-expressed in SF21 insect cells infected with baculoviruses containing the bacmids K1999, K2124, K2126, K2209, K2123, K2212, K2204 or K1721, respectively. Expression of the different bait proteins is indicated (+: untagged protein expressed; T:TAP-tagged; F: FLAG-tagged; *: bait protein). Purifications were done from lysates of 5×10^7^ infected insect cells with either IgG-coupled beads (Lanes 4–6 and 8) or with anti-FLAG affinity matrix (Lanes 1–3 and 7) and eluted with TEV protease or FLAG peptide, respectively. Half of the elution material was analyzed with SDS-PAGE and MS analysis (top panel) (**D**) Cell extracts and purified samples of the SF21 insect cells described in Fig. 3C were analyzed by SDS-PAGE and WB. Indicated protein content was identified by MS analysis (Coomassie staining panel); samples from cell extracts, elution (10%) and resin before elution, were analyzed by WB using the anti-FLAG antibody.

As described above, co-expression of yeast tUTP proteins in insect cells led to the reconstitution of a fully-assembled, heptameric tUTP complex. Moreover, the results of these experiments indicate the formation of a tUTP pentamer composed of Utp4, Utp5, Utp8, Utp9 and Utp15. To test whether formation of a stable tUTP pentamer is possible in the absence of Utp10 and Nan1, a baculovirus encoding the five proteins of the tUTP pentamer was used to infect insect cells. Utp4-TAP affinity purification and subsequent analyses of the eluting proteins by SDS-PAGE and MS (Fig. 3A) confirmed the co-purification of all co-expressed proteins (Fig. 3A, Lane 1). When Utp4-TAP affinity-purified complexes were subjected to a second purification using Utp15-FLAG as the bait protein, all co-expressed components were present in the eluate (Fig. 3A, Lane 2). Likewise, all components of the purified tUTP complex were stained with similar intensity by Coomassie Blue, and co-eluted from the gel filtration column with an apparent molecular weight of approximately 600 kDa (Fig. 3B). Taken together, these results confirm that the formation of the tUTP pentamer is independent from the presence of Nan1 and Utp10.

Although, previous yeast two hybrid analyses suggested a direct interaction between Nan1 and Utp10 [14], the experiments described in this work provide evidence for a weak association between these two proteins and the tUTP pentamer. In order to clarify this interaction *in vitro,* the yeast proteins Utp10 and Nan1-FLAG were co-expressed in insect cells. Predictably, Nan1-FLAG affinity purification from corresponding insect cell extracts efficiently enriched both proteins (Fig. 3C, Lane 1). These data indicate that a complex of Nan1 and Utp10 can be formed in the absence of other tUTP components through direct interactions.

In order to study the protein interactions responsible for the formation of the tUTP pentamer, insect cells were infected with viral genomes containing different combinations of yeast tUTP pentamer components. The resulting cell extracts were used for affinity purification of the indicated bait proteins, and the eluates from the different purifications were analyzed by SDS-PAGE and MS analysis (Fig. 3C and 3D).

First, Utp4-TAP was co-expressed with Utp5 and Utp15-FLAG. Utp4-TAP affinity purification from cellular extracts confirmed the co-purification of all three proteins (Fig. 3C, Lane 4). Moreover, the MS identification of both Utp4 and Utp5-TAP in the eluate from the Utp4-TAP affinity purification of co-expressed Utp4-TAP and Utp5 (Fig. 3C, lane 8) suggests a direct interaction between Utp4 and Utp5. In parallel, Utp15-FLAG purification from cellular extracts, in which Utp5 and Utp15-FLAG were co-expressed, showed the presence of both proteins in the eluate, indicating the direct interaction between both proteins (Fig. 3C, Lane 2). Altogether, these data indicated that the tUTP pentamer contains a trimeric building block made of Utp4 and a Utp5-Utp15 heterodimer. Significant amounts of Utp8-FLAG and Utp9 were detected in the eluate by MS analysis (Fig. 3C, Lane 7) when both proteins were co-expressed and Utp8-FLAG affinity-purified. This result indicates a direct interaction of Utp8 and Utp9, which is in agreement with published data showing an independent association of these proteins from the formation of the tUTP/UTP A subcomplex [21]. To further elucidate whether the formation of the tUTP pentamer involves interactions of Utp4 with the Utp8-Utp9 heterodimer, Utp4-TAP was co-expressed with Utp8-FLAG and Utp9. When Utp4-TAP was used as the bait protein, Utp8 and Utp9 were not detected in the respective eluates (Fig. 3C, Lane 6). Only a weak signal corresponding to Utp8-FLAG was observed by WB analysis prior to the TEV elution, indicating some association between Utp4 and Utp8 with the resin (Fig. 3D, Beads panel, Lane 6). A possible explanation for the apparent low co-purification of Utp8 and Utp9 with Utp4-TAP might be an insufficient expression level of these two proteins in the respective insect cells. Nevertheless, WB detection of Utp8-Flag and MS detection of Utp9 in corresponding insect cell extracts argued against this possibility (Fig. 3D Coomassie and Cell Extracts Panel, Lanes 3–7). We conclude that the weak interaction of Utp4 with the Utp8-Utp9 dimer is stabilized by the presence of the Utp5-Utp15 dimer in the tUTP pentamer.

In summary, these experiments showed that the tUTP complex is made of several building blocks, which can form independently of other yeast components. They include a Utp10-Nan1 dimer and a pentameric complex made of a Utp8-Utp9 dimer and Utp4 bound to a dimer of Utp5 and Utp15.

### Identification of the building blocks of the yeast UTP B subcomplex

**Figure 4:**
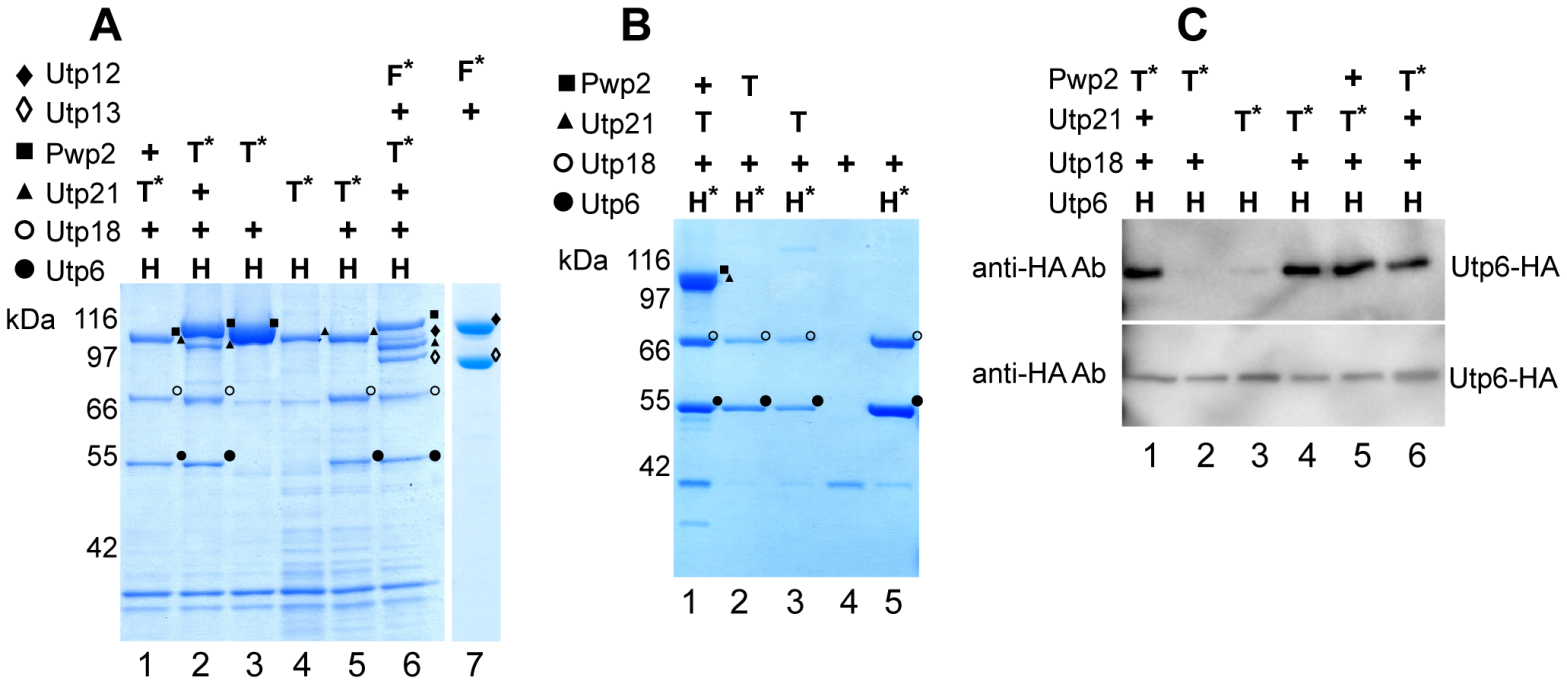
Identification of different UTP B building blocks. Tagged proteins were purified from cell extracts containing different UTP B components in one or two step affinity purifications. Correct identification by MS analysis of the corresponding protein is indicated as Pwp2, ▪; Utp6, •; Utp12, ♦; Utp13, ◊; Utp18, ○ and Utp21, ▴. Expression of the tagged proteins is indicated as +: untagged protein expressed; T:TAP-tagged; F: FLAG-tagged; *: bait protein. (**A**) Combinations of the indicated proteins were co-expressed in SF21 insect cells infected with baculoviruses containing the bacmids K2137, K1987, K2134, K2135, K2136, K1991 and K1978. The bait proteins were purified from lysates of 5×10^7^ infected insect cells with IgG-coupled beads and eluted with TEV protease (Lanes 1–6) or with anti-FLAG affinity beads and elution with FLAG peptide (Lane 7). Samples of the elution were analyzed with SDS-PAGE and MS analysis. (**B**) Combinations of the indicated proteins were co-expressed in SF21 insect cells infected with baculoviruses containing the bacmids K2137, K2134, K2136, K2138 and K2139. Expression of the tagged proteins is indicated. The bait proteins were purified from lysates of 5×10^7^ infected insect cells with anti-FLAG affinity matrix and eluted with the FLAG peptide. Samples of the elution were analyzed with SDS-PAGE and MS analysis. Note that a band compatible with the size of Utp4-TAP is observed in Lane 3 but was not possible to characterize by MS analysis. (**C**) Combinations of the indicated proteins were co-expressed in SF21 insect cells infected with baculoviruses containing the bacmids K1991, K2134, K2135, K2136, K2137 and K1987. The bait proteins were purified from lysates of 5×10^7^ infected insect cells with IgG-coupled beads and eluted with TEV protease. Aliquots of the elution (upper panel) or of the corresponding cell lysate (lower panel) were analyzed by WB with anti-HA antibody. The corresponding co-expressed proteins are indicated at the top of the figure.

The experiments for the reconstitution of the UTP B subcomplex suggested a stable, tetrameric module consisting of Pwp2, Utp6, Utp18 and Utp21, which interacts with Utp12 and Utp13 (Fig. 2). To assay whether the tetrameric core module can be reconstituted independently of Utp12 and Utp13, insect cells were infected with two different viral genomes encoding the yeast proteins Pwp2, Utp6, Utp18 and Utp21 where either Pwp2 or Utp21 were TAP-tagged. Affinity purification of both Pwp2-TAP and Utp21-TAP from the corresponding cell extract, resulted in co-purification of all four proteins (Fig. 4A, Lanes 1 and 2) and confirmed the existence of a stable, tetrameric building block. This complex could also be isolated by anti-HA affinity purification from cells expressing HA-Utp6, Utp21-TAP, Utp18 and Pwp2 (Fig. 4B, Lane 1). Interestingly, co-expression of Utp12-FLAG and Utp13 lead to the detection of both proteins after FLAG affinity purification (Fig. 4A, Lane 7). Thus, these results identified a tetrameric core-complex composed of Pwp2, Utp6, Utp18, and Utp21 with the associated heterodimer, Utp12-Utp13, as building blocks of the UTP B complex.

To better dissect the architecture of the UTP B core-complex, insect cells were independently transfected with different combinations of yeast UTP B core complex components. When Utp21-TAP, Utp6-HA, and Utp18 were co-expressed, Utp21-TAP affinity purification revealed all three proteins in the eluate (Fig. 4A, Lane 5). On the other hand, after Utp6-HA affinity purification, both Utp6 and Utp18 were also identified in the corresponding eluates (Fig. 4B, Lane 3). These results indicated the formation of a trimeric building block of the UTP B core complex, made of Utp21, Utp6 and Utp18. Co-expression of only Utp6-HA and Utp21-TAP, and subsequent affinity purification, did not result in any detectable co-purification (Fig. 4C, Lane 3, upper panel). In contrast, co-expression of Utp6-HA and Utp18, followed by Utp6-HA affinity purification, showed the presence of both proteins in the eluate, indicating direct interaction between these two proteins (Fig. 4B, Lane 5).

Finally, Pwp2-TAP affinity purification from the co-expression of Pwp2-TAP, Utp6-HA, and Utp18 did not yield detectable amounts of either Utp6 or Utp18 in the purified fraction (Fig. 4A, Lane 3). Utp6-HA expression levels were also verified by WB analysis (Fig. 4C, lower panel), suggesting a similar expression level in all cellular extracts. Interestingly, MS analysis of an HA affinity purification from the same cellular identified Utp6 and Utp18 but not Pwp2 in the eluate (Fig. 4B, Lane 2). These results are in agreement with a direct interaction between Utp6 and Utp18 proteins, but they argue against a stable interaction of the Utp6-Utp18 heterodimer with Pwp2. Consequently, we conclude that Utp21 is required to recruit Pwp2 to the UTP B core complex.

In summary, these experiments identified several autonomous building blocks of the UTP B subcomplex. First, the observation of a stable tetrameric core-complex formed by proteins Pwp2, Utp21, Utp6 and Utp18, which could be assembled in the absence of the Utp12-Utp13 dimer. Furthermore, our data suggest a Pwp2-independent formation of the trimeric building block Utp21-Utp6-Utp18, in which Utp18 is required for stable association of Utp21 with the heterodimer Utp6-Utp18. In turn, Utp21 appears to mediate the association of Pwp2 with the Utp21-Utp6-Utp18 heterotrimer.

## Discussion

**Figure 5:**
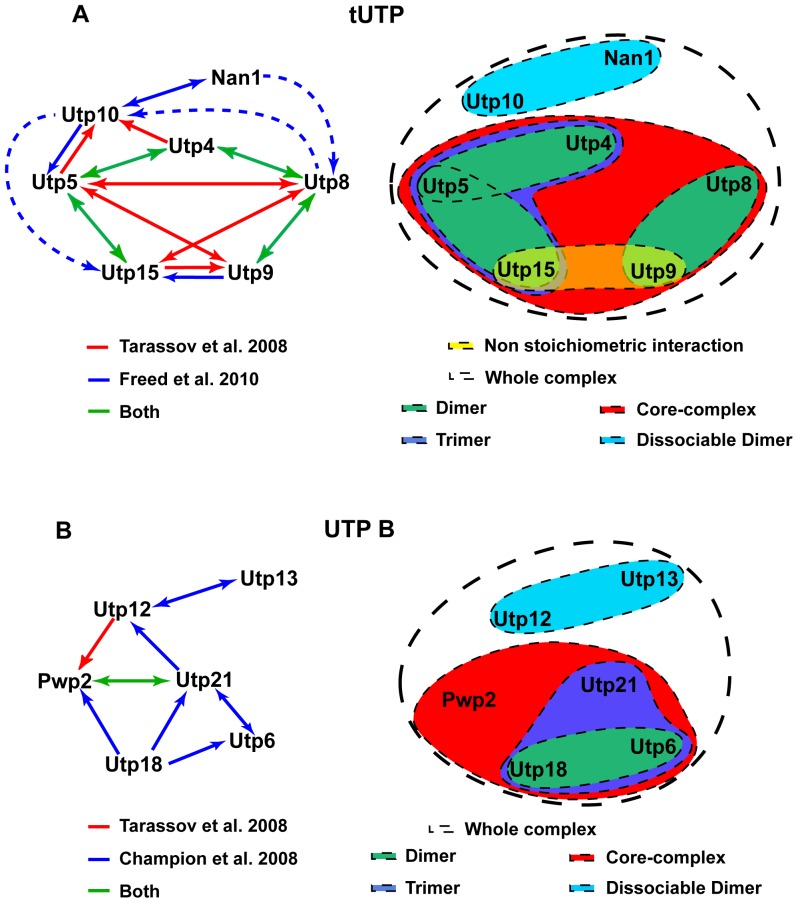
Refined model of tUTP and UTP B architecture. Binary interactions observed by protein-fragment complementation assay [13] (red line), yeast two hybrid assay [12], [13] (blue line) or both (green line) are depicted for tUTP (**A**) and UTP B (**B**) components. Arrows point from prey to bait proteins. Building blocks observed in the present study are grouped by solid surfaces for tUTP (**A**) and UTP B (**B**) subcomplexes. Loose interaction (yellow surface); whole complex (white surface); Dimer (green surface); Trimer (purple surface); core-complex (red surface); dissociable dimer (blue surface).

In previous studies, the yeast subcomplexes tUTP/UTP A and UTP B have been described in terms of functionality and protein composition [7], [8], [10], [11]. Moreover, binary interactions between their protein components have been identified by several approaches [12]–[14] (Fig. 5A and B, left side). The present study took advantage of a heterologous expression system to identify the relevant protein building blocks leading to the formation of these subcomplexes.

The yeast subcomplexes tUTP and UTP A were suggested to be related [8], [10], since their proposed composition only differs in the UTP A-specific protein, Pol5, and the tUTP-specific protein, Utp5 [7], [8], [10]. In this work, co-expression of candidate tUTP subunits allowed the isolation of a fully reconstituted tUTP complex. Accordingly, our experiments provide biochemical evidence that the functionally related tUtps form a protein complex, as suggested previously [8], [10]. Co-expression of all candidate UTP B components also enabled reconstitution of the expected subcomplex with a molecular size compatible with a hexameric protein complex.

Expression of only subsets of tUTP or UTP B components lead to the identification of smaller protein complexes, which were not predicted by the existent binary data. Our results suggest they reflect architectural building blocks of the yeast tUTP and UTP B subcomplexes (Fig. 5A and B, right side). The combined data (Fig. 5) point to some shared architectural features of tUTP and UTP B. In both subcomplexes either a pentameric tUTP or a tetrameric UTP B core-complex interacts with a more loosely associated dimer, Nan1-Utp10 and Utp12–Utp13, respectively. This fact could indicate a more peripheral position of the aforementioned heterodimers. Moreover, the expression of specific combinations of subcomplex components argues for a central role of Utp4 in the formation of the tUTP core-complex and of Utp21 in the formation of the UTP B core-complex. Indeed, recent structural analysis of Utp21 supports this notion by indicating two binding platforms which might establish simultaneous interactions with the Utp6-Utp18 dimer and Pwp2 [22]. Future studies are necessary to delineate how the central role of Utp4 in the formation of the tUTP is relayed in the mammalian orthologue due to the absence of proteins Utp8 and Utp9 [9].

Besides their likely role as architectural units, the tUTP and UTP B building blocks identified in this work might also represent assembly or disassembly intermediates of these subcomplexes. Currently, little is known of the formation of the tUTP and UTP B subcomplexes *in vivo*. These complexes might be formed on nascent pre-rRNA or assembled in the cytoplasm and enter the nucleus/nucleolus as preformed complexes. In this regard, ribosome production in insect cells should be highly downregulated after viral infection [23]. Thus, tUTP and UTP B formation should occur independent of ongoing ribosome biogenesis. Moreover, both protein subcomplexes are produced in the absence of any other yeast factor, including yeast pre-rRNA, which indicates an assembly mechanism mainly triggered by the intrinsic affinities of the subcomplex components. Still, auxiliary factors/chaperones conserved among eukaryotes might facilitate subcomplex formation. In any case, we consider as a possibility that *in vivo* assembly of the yeast subcomplexes tUTP and UTP B might involve transient formation of the building blocks identified in this work.
